# Supplementary material for: Chromothripsis during telomere crisis is independent of NHEJ, and consistent with a replicative origin
Source: Genome Res. 2019 May;29(5):737–49. doi: 10.1101/gr.240705.118 (PMC6499312; doi:10.1101/gr.240705.118)
Supplement: Supplemental Material [file supp_gr.240705.118_Supplemental_file_1.zip › contigs/annotated_contigs/DB51/contig.2.DB51_length_791_mean_cov_8.43109987358.docx]

**DB51_length_791_mean_cov_8.43109987358**

```
AACCATTGTTCTGCTTTATGGTACAATAGATTAGTTTGCATTTTCTGCAATTTTACATAAAAAGAATCATAGGGTCTGTACATATCTTT
 >chr3:85947633-85948078 + E=4e-251
CTTTTTTTTTTTTGAGTCTCAACATAAGTATTTGAGACTCATTCATGTTGTTGCATGTACATAGTTTCTTCTCTGTTTTCTTGCTGAAT

AATATTCTATTGTATGGATACATACGCCACAATTAGTTACCTGTTTACCCAGTGATGGACATTCAGGTTGTTTATAGTTTGCAGATATT

ATAGTAAAGCTACTATGAACATTTGTGGGTACAATTTTGTATGGCCATTTGTCTTCATTTTTCTTAGGCAAATGCCTAGATGCAGACTA

GCTGGATTATATGGTAGCCATATATTTAATTTTTTAAAGGCCTTGCCATTTTCCAAAATGTTTGTACTACTTCATAAAATCCAA|TTCA
                                                                                     >ch
|AACATCAAGAAAAGGTTGCCTCCTAAGATACTAAATTACTTCCTTTGAGTAGTATTATAAAGATGAATTCTCTAAACCCCAAAGTATC
r3:86006074-86006425 + E=3e-196
ACCAGTATAACTTCTATTTTAATATGGGTCTTATGTGACTTTGTGGAAAGGGCAAGATTTCTACATTGCTACAAATCATAGTTTTATTT

TATCATTTTTTGCTGCTTCAAGAGATGTTAACGATGTACATAGCACAAGACAATCTAGCCTGTTATAAAATAAGAATTGGTAAATTCAA

ATAGCAGTGAAAAGATTTAGGATAAATGAAGCATGTTGAGAACTATTAATAATTTGGCTTTTATTAAGGATACTAGGGGAG
```
